# Study on the Potential Toxicity of a Thymoquinone-Rich Fraction Nanoemulsion in Sprague Dawley Rats

**DOI:** 10.3390/molecules18077460

**Published:** 2013-06-26

**Authors:** Zaki Tubesha, Mustapha Umar Imam, Rozi Mahmud, Maznah Ismail

**Affiliations:** 1 Laboratory of Molecular Biomedicine, Institute of Bioscience, Universiti Putra Malaysia, 43400 Serdang, Selangor Darul Ehsan, Malaysia; 2 Department of Nutrition and Dietetics, Faculty of Medicine and Health Sciences, Universiti Putra Malaysia, 43400 UPM, Serdang, Selangor, Malaysia; 3 Department of Imaging, Faculty of Medicine and Health Sciences, Universiti Putra Malaysia, 43400 UPM, Serdang, Selangor, Malaysia

**Keywords:** Sprague-Dawley rats, nanoemulsion, thymoquinone rich fraction, acute oral toxicity, histopathology, hematology

## Abstract

Toxicological studies constitute an essential part of the effort in developing an herbal medicine into a drug product. A newly developed thymoquinone-rich fraction nanoemulsion (TQRFNE) has been prepared using a high pressure homogenizer. The purpose of this study was to investigate the potential acute toxicity of this nanoemulsion in Sprague Dawley rats. The acute toxicity studies were conducted as per the OECD guidelines 425, allowing for the use of test dose limit of 20 mL TQRFNE (containing 44.5 mg TQ)/kg. TQRFNE and distilled water (DW) as a control were administered orally to both sexes of rats on Day 0 and observed for 14 days. All the animals appeared normal, and healthy throughout the study. There was no observed mortality or any signs of toxicity during the experimental period. The effects of the TQRFNE and DW groups on general behavior, body weight, food and water consumption, relative organ weight, hematology, histopathology, and clinical biochemistry were measured. All the parameters measured were unaffected as compared to the control (DW) group. The administration of 20 mL TQRFNE /kg was not toxic after an acute exposure.

## 1. Introduction

Drug efficacy and bioavailability can be severely limited by poor aqueous solubility and some drugs also show side effects due to their poor solubility [1]. Moreover, it is widely recognized that up to 60% of new drugs in the developmental stage are water insoluble [2]. Therefore, the improvement of drug solubility and thereby its bioavailability remains one of most challenging aspects of drug development process [1]. In the past few decades, considerable attention has been focused on the development of novel drug delivery systems for herbal drugs [2]. However some limitations during consumption of herbal extracts or plant bioactives like instability in highly acidic pH and liver metabolism led to drug levels below therapeutic concentration in the blood resulting in less or no therapeutic effect [3]. Hence, encapsulation of plant extracts or its bioactives minimizes their degradation or presystemic metabolism, and serious side effects by accumulation of drugs to the non-targeted areas and improves the ease of administration in the pediatric and geriatric patients [4].

A wide variety of drug carriers including nanoemulsions, microemulsions, liposomes, solid lipid nanoparticles, microspheres, and self-microemulsifying drug-delivery systems have been studied to improve the therapeutic efficacy of hydrophobic drugs/nutraceuticals by enhancing the bioavailability and tissue-targeting ability [5]. Lipid nanoemulsions containing oil from medicinal plants or hydrophobic drugs have been shown to improve drug solubility, reduce side effects of various potent drugs, increase the bioavailability of drugs, and to prolong the pharmacological effects in comparison to conventional formulations such as conventional emulsions [6]. Hence, nanoemulsions can be used as novel formulations in many areas including pharmaceutics, cosmetics, food technology, and other sectors [7]. Despite the beneficial effects of the nanocarriers on living cells, undesirable effects may occur. Hence, before marketing a new nanocarrier, extensive toxicity tests are required before it is deemed "safe" for marketing [8]. When sufficient toxicological data from *in vitro* and *in vivo* experiments are available, the pharmaceutical then may be applied to a human population. Therefore, data obtained from such tests are then used to extrapolate the doses and effects on humans [9]. Determination of acute oral toxicity is usually an initial screening step in the assessment and evaluation of the toxic characteristics of newly developed compounds [10].

*Nigella sativa* oil and its main active constituent, thymoquinone (TQ), are extensively reported to exhibit protective effects against many diseases largely attributable to its high antioxidant activity [11,12]. However, thymoquinone-rich fraction (TQRF) and TQ are lipophilic substances with limited absorbance in living system. Hence, nanoemulsions from TQRF and TQ were developed in attempt to increase their absorbance and bioavailability. Regarding the safety of *N. sativa*; its seed powder did not produce any toxic effects at very high doses (28 gm/kg orally) in rabbits; its oil was also safe when given orally to rats (LD_50_ of 28.8 mL/kg); and oral TQ was also found to be safe (LD_50_ of 2.4 g/kg) [13]. However, there are toxicological concerns and ethical issues that come with nanomedicine and they have to be addressed alongside the benefits. Moreover, nanotoxicology is now becoming a very important field in view of the unique physicochemical properties of nanomaterials that may lead to adverse biological effects on occupational, environmental and consumer matters [14]. Hence, despite the wide margin of safety of *N. sativa* oil, the toxicity study of newly developed TQRF nanoemulsion (TQRFNE) is very important.

## 2. Results and Discussion

### 2.1. Clinical Signs and Necropsy Findings

This study was designed to assess the acute oral toxicity of TQRFNE which was administered by oral gavage to male and female rats. No deaths or obvious clinical signs were found in any groups throughout the experimental period. Physical observation of the treated rats throughout the study indicated that none of them showed signs of toxicity such as skin, fur, eyes, mucus membrane and behavioral changes, tremors, salivation, diarrhea, sleep and coma. Normal body weight gains were observed during the study period compared to the control group. No abnormal gross findings were observed in the necropsies of any of the animals. 

### 2.2. Body Weight, Food and Water Consumption during the 14 Days

**Figure 1:**
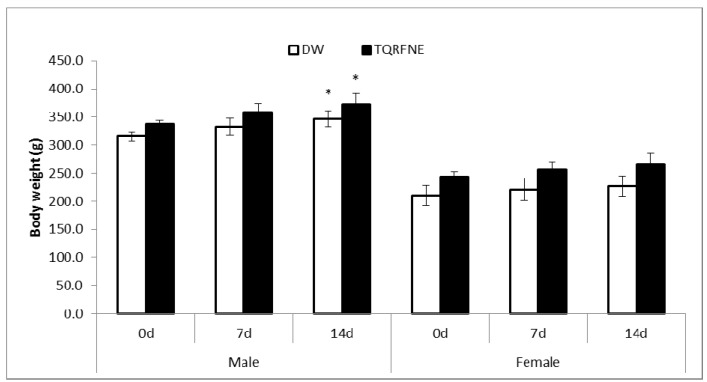
Effects of oral administration of thymoquinone-rich fraction nanoemulsion (TQRFNE) and distilled water (DW) on body weights of male and female rats (n = 5). For each gender P values < 0.05 were considered as significant using one way ANOVA followed by Least Significant Difference (LSD). Asterisks on bars of 7^th^ and 14^th^ days denote significant difference compared to day 0 in each gender.

**Figure 2:**
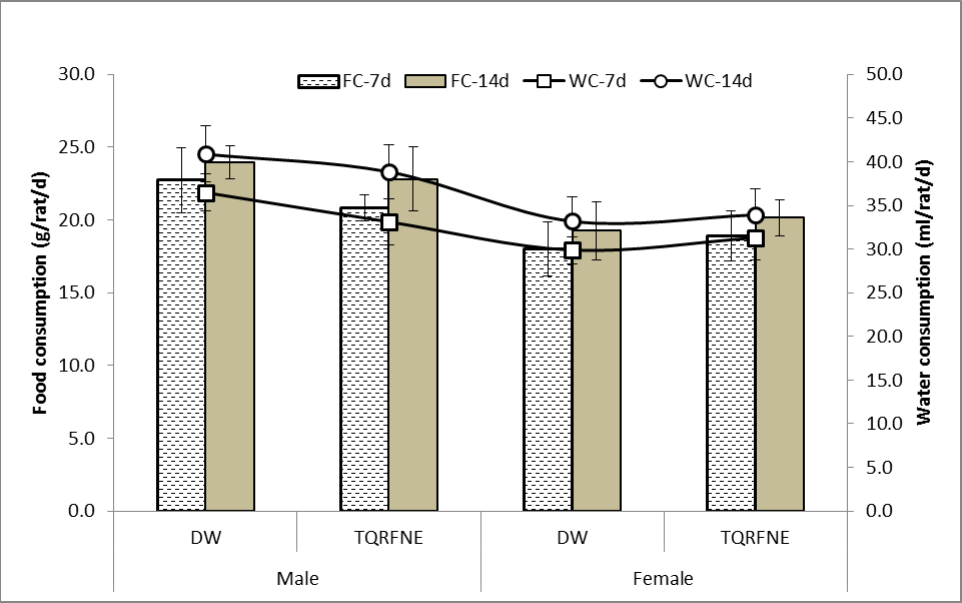
Effects of oral administration of thymoquinone-rich fraction nanoemulsion (TQRFNE) and distilled water (DW) on daily food (g/rat/d) and water (ml/rat/d) consumption of male and female rats (n = 5). Values are expressed as mean ± SD. P values < 0.05 were considered as significant using one way ANOVA followed by Least Significant Difference (LSD). For each gender, there was no significant difference (*p* ≥ 0.05) compared to the control group (DW). FC: Food consumption, WC: Water consumption.

The body weights of the TQRFNE and control rats are shown in Figure 1. There were gradual increases in body weight of TQRFNE and control rats. After the first week of the study, the body weight increases of all groups were not significant for both sexes compared to day 0; similar results were also shown for female rats after 2 weeks (day of sacrifice). On the other hand, both male groups (TQRFNE and control) exhibited a significant body weight increase after 2 weeks. At the end of the study, the percentages increase in body weight for male of treatment rats were found to be 9.83 and 10.37%, and for female rats 7.89% and 8.87% of distilled water (DW) and TQRFNE, respectively. The food and water consumption of the treated rats were also not significantly different compared to the control rats measured throughout the study (Figure 2). The progressive increase in body weights of rats during the study period may indicate the improvement in the nutritional state of the animals. The growth response effect could be as a result of increased food and water intake.

### 2.3. Relative Organ Weights (ROW) of Male and Female Rats

For prediction of basal metabolism, use of organ weights and ROW seem to be promising in safety and toxicity assessment [15]. There were no significant (*p* > 0.05) differences between ROW of treated and control rats (Table 1). The progressive increase in body weights and organ weights of rats during the study period may indicate the improvement in the nutritional state of the animals. The growth response effect could be as a result of increased food and water intake. Similar findings have been conducted [15,16,17], and the reported values for ROWs of Sprague dawley rat strains support those of the current data sets. However, age and strains of experimental animals can significantly affect ROW values.

Values are expressed as mean ± SD of 5 rats in each group. In each row and each gender P values < 0.05 were considered as significant using one way ANOVA followed by Least Significant Difference (LSD). No significant differences were observed in any parameter compared to control (DW).

**Table 1:** Effects of oral administration of thymoquinone-rich fraction nanoemulsion (TQRFNE) and distilled water (DW) on mean terminal body weight (bw) and relative organ weights of male and female rats during acute toxicity study

Parameters	Male		Female	
	DW	TQRFNE	DW	TQRFNE
**Fasted bw (g)**	346 ± 14	373 ± 19	227 ± 17	265 ± 20
**Liver**	3.10 ± 0.18	3.23 ± 0.22	2.78 ± 0.15	2.93 ± 0.21
**Kidneys**	0.65 ± 0.05	0.70 ± 0.07	0.57 ± 0.04	0.60 ± 0.04
**Spleen**	0.23 ± 0.02	0.24 ± 0.01	0.20 ± 0.02	0.21 ± 0.01
**Heart**	0.28 ± 0.01	0.31 ± 0.04	0.25 ± 0.02	0.25 ± 0.01
**Lungs**	0.54 ± 0.03	0.54 ± 0.04	0.48 ± 0.04	0.50 ± 0.07
**Brain**	0.63 ± 0.05	0.64 ± 0.07	0.53 ± 0.03	0.52 ± 0.05
**Gonads (Testes/Ovaries)**	0.72 ± 0.07	0.68 ± 0.08	0.65 ± 0.04	0.65 ± 0.06
**Stomach (empty)**	0.40 ± 0.06	0.44 ± 0.06	0.36 ± 0.04	0.37 ± 0.04
**Gut (empty)**	2.88 ± 0.35	2.88 ± 0.34	2.59 ± 0.16	2.75 ± 0.35

### 2.4. Hematology

There is a correlation of toxicity in hematological, gastrointestinal and cardiovascular adverse effects between animals and humans. In addition, hematological indices in animals are important to determine the toxicity risk since the changes in the blood system have a higher predictive value for human toxicity [18]. In this study, hematological indices were used to assess the toxicity of TQRFNE in Sprague Dawley rats. The hematological parameters of experimental and control rats are presented in Table 2. No significant alterations were seen in the hematological profile of any of the experimental groups studied herein.

**Table 2:** Effects of oral administration of thymoquinone-rich fraction nanoemulsion (TQRFNE) and distilled water (DW) on hematological parameters of male and female rats

Parameters	Male		Female	
	DW	TQRFNE	DW	TQRFNE
**WBC (10^3^/µL)**	17.6 ± 1.7	17.5 ± 1.3	13.2 ± 1.1	12.1 ± 1.2
**RBC (10^6^/µL)**	8.0 ± 0.5	8.0 ± 0.6	7.0 ± 0.2	6.8 ± 0.3
**HGB (g/dL)**	14.3 ± 0.7	14.6 ± 1.0	13.4 ± 0.3	13.9 ± 0.80
**HCT %**	44.6 ± 2.4	43.3 ± 1.1	41.9 ± 2.4	40.5 ± 3.0
**MCV (FL)**	56.3 ± 1.1	56.7 ± 0.7	58.8 ± 0.7	60.9 ± 1.8
**MCH (Pg)**	18.0 ± 0.2	18.2 ± 0.57	19.4 ± 0.05	20.2 ± 0.5
**MCHC (g/dL)**	32.0 ± 0.4	32.3 ± 0.45	33.4 ± 0.37	34.4 ± 0.8
**PLT (10^3^/µL)**	1108.0 ± 125	1137.8 ± 12	1109.2 ± 55	1292.6 ± 137
**LYM# (10^3^/µL)**	11.4 ± 1.2	12.9 ± 1.2	10.2 ± 0.6	9.3 ± 1.5
**PDW (fL)**	9.5 ± 0.5	9.0 ± 0.879	9.0 ± 0.22	8.6 ± 0.47
**MPV (fL)**	7.7 ± 0.2	7.4 ± 0.48	7.6 ± 0.21	7.4 ± 0.21
**P-LCR %**	7.9 ± 0.2	7.6 ± 1.0	8.3 ± 0.92	7.9 ± 0.47

Values are expressed as mean ± SD of five rats in each group. In each row and each gender P values < 0.05 were considered as significant using one way ANOVA followed by Least Significant Difference (LSD). No significant differences were observed in any parameter compared to control (DW). WBC-white blood cell, RBC-red blood cell, HGB-hemoglobin, HCT-hematocrit, MCV-mean corpuscular volume, MCH-mean corpuscular hemoglobin, MCHC-mean corpuscular hemoglobin concentration, PLT-platelet, LYM-lymphocyte, PDW-platelet distribution width, MPV-mean platelet volume, P-LCR-platelet larger cell ratio.

### 2.5. Liver and Kidney Functions Tests

Liver and kidney function tests are important parameters in determining the safety of functional ingredient or final product [19]. In a toxic environment the blood level of alkaline phosphatase [ALP], alanine amino-tranferase (ALT), asparatae amino-tranferase (AST), gamma glutamyl transpeptidase (GGT), urea (URE), and creatinine (CREA) are known to significantly increase [20,21]. The first four enzymes are reliable indices of liver toxicity and the last two parameters for kidney toxicity [22]. All these parameters in the study (Table 3) showed no appreciable increase in the treated animals except AST in the male rats treated with TQRFNE (111.0 ± 13 mM compared to 93.9 ± 7 mM in control group). AST and ALT are classical enzymes assayed in liver function tests. Increases in the levels of these enzymes above normal are reliable indices of liver toxicity or altered integrity of cellular membrane and cell death [23]. However, AST is not a highly specific indicator for liver injury because it is found in other tissues like the heart, muscles, kidney, and brain, unlike ALT which is found largely in the liver [22]. In addition, the results of the female TQRFNE group were normal compared to control group, so the slightly higher AST may not be considered toxicologically relevant. Hence, this is the first report on the acute oral toxicity study of TQRFNE indicating that it does not have significant toxic effects on the liver and kidneys.

**Table 3:** Effects of oral administration of thymoquinone-rich fraction nanoemulsion (TQRFNE) and distilled water (DW) on liver and kidney function tests of male and female rats.

Parameters	Male		Female	
	DW	TQRFNE	DW	TQRFNE
**ALP (mM)**	123.2 ± 32	112.9 ± 13	71.7 ± 14	64.2 ± 14
**ALT(mM)**	40.7 ± 7	41.2 ± 4	40.8 ± 5	38.3 ± 2
**AST(mM)**	93.9 ± 7	111.0 ± 13*	89.4 ± 13	101.7 ± 10
**GGT(mM)**	7.9 ± 1	7.6 ± 1	7.3 ± 0.9	7.5 ± 0.7
**CREA(mM)**	33.3 ± 3	34.1 ± 7	34.3 ± 5	36.2 ± 5
**UREA(mM)**	5.7 ± 0.7	5.4 ± 0.9	5.1 ± 0.3	5.5 ± 0.8

Values are expressed as mean ± SD of 5 rats per group. In each row and each gender P values < 0.05 were considered as significant using one way ANOVA followed by Least Significant Difference (LSD). * denote significance compared to control group (DW). ALP-alkaline phosphatase, ALT-alanine amino-tranferase, AST-asparatae amino-tranferase, GGT-gamma glutamyl transpeptidase, URE-urea, CREA-creatinine.

### 2.6. Histopathology

**Figure 3:**
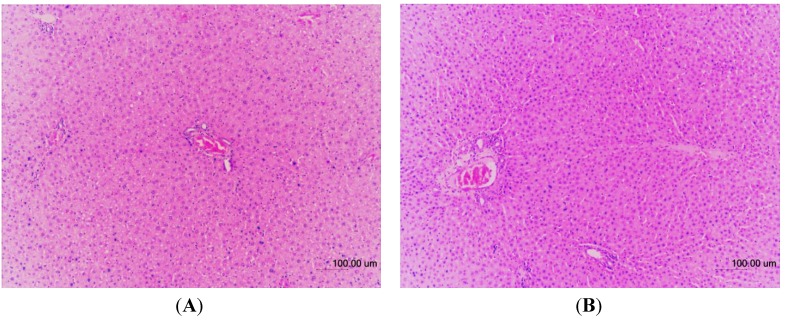
Hematoxylin and Eosin-stained sections of male liver tissues from control (**A**) and TQRFNE (**B**) treated groups. No significant damage was detected in both groups.

**Figure 4:**
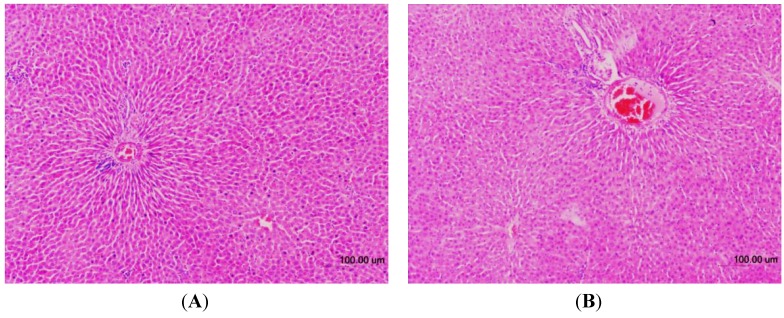
Hematoxylin and Eosin-stained sections of female liver tissues from control (**A**) and TQRFNE (**B**) treated groups. No significant damage was detected in both groups.

The histopathological examinations of the liver tissues from male and female rats were performed to further confirm whether or not the tissues had been damaged (Figure 3, Figure 4, respectively). No significant histopathological changes in the liver tissues of the experimental animals were observed. Compared to control groups all TQRFNE groups showed normal hepatic architecture with portal triad surrounded by the hepatocytes. Narrow sinusoids open freely into the portal vein. Gross and histopathological examinations further confirmed that the administration of TQRFNE (20 mL/kg) for both sexes of Sprague Dawley rats did not cause any evidence of liver tissue damage as compared to control groups. Therefore, the results suggest that the administration of 20 mL TQRFNE /kg is not toxic after an acute exposure.

Nanoemulsions have a higher solubilization capacity than simple micellar solutions and their thermodynamic stability offers advantages over unstable dispersions such as conventional emulsions [24]. Therefore, TQRFNE may associate with lymph lipoprotein in the enterocytes and gain access to the intestinal lymphatics, effectively bypassing the liver and gaining access to the systemic circulation via the thoracic lymph duct. In addition, we speculated that the presence of TQRF oil in the nanoemulsion would probably enhance oral absorption based on structural similarities to chylomicrons. In an earlier study, a group of 10 mice were given *N*. *sativa* fixed oil at the dose of 10mL/kg p.o, approximately containing 10 times higher concentration of TQRF compared to that used in the current study. The animals were observed for gross effects and mortality for 15 days. There were no adverse effects or mortality during the observation period following the administration of *N. sativa* fixed oil in mice [25]. In addition, Zaoui,* et al.* [26] investigated the acute toxicity of the *N. sativa* fixed oil in mice through determination of LD_50_ values. They found that, LD_50_ of N. sativa fixed oil was 28.8 mL/kg bw. p.o. (approximately higher than our TQRF content by 28.8 fold) and 2.06 mL/kg bw. i.p., respectively. However, concentrations of TQRF much lower than these were reported to produce functional effects. In fact, TQRF content similar to that in our TQRFNE showed significant improvements in metabolic outcome [11,27,28]. In view of the increased solubility and bioavailability of nanoemulsions [29,30], we hypothesized that our TQRFNE, containing a similar amount of TQRF reported to be functionally active, could potentially produce effects at much lower concentrations without the risks of toxicity.

## 3. Experimental

### 3.1. Materials

All the chemicals used in this study were of analytical grade; TQ, Glycerol trioleate (Triolein, TR) and Polysorbate 80 (Tween 80) were purchased from Sigma (Sigma-Aldrich Co., St. Louis, MO, USA). Formalin, cassettes, Haematoxylin and Eosin (H & E) were purchased from Fisher Scientific (Loughborough, Leicestershire, UK). Clinical chemistry kits for serum biochemistry parameters (ALP, ALT, AST, GGT, URE, and CREA) were purchased from Randox Laboratories Ltd (Crumlin, County Antrim, UK). Double distilled water was used for the preparation of emulsions. Ethanol, xylene, and paraffin were purchased from Merck (Darmstadt, Germany). 

### 3.2. Production of TQRFNE

TQRFNE was prepared by homogenizing 5% of TQRF oil (contains 4.45% TQ) with 95% aqueous phase (2% Tween-80, and 93% DW) using two sequential homogenization methods. The first method involved homogenizing the solutions in an Ultra-Turrax T25 (IKA, Staufen, Germany) for 3 min at 13000 rpm. The second method involved subjecting the previously prepared emulsion to a high-pressure homogenization process using a bench top high–pressure homogenizer (Stansted Fluid Power, Ltd., Essex, UK) at a pressure of 800 bar for five cycles to produce the TQRFNE. To avoid degradation of bioactives, the nanoemulsion was cooled to less than 25 °C using an ice bath after each homogenization cycle. The final concentration of TQ in the nanoemulsion was determined to be 2.25 mg/mL (44.5 mg TQ/20 mL TQRFNE).

### 3.3. Experimental Animals

Male and female Sprague Dawley rats (n = 20, 10 ♂ and 10 ♀) at 12 weeks of age were used in the study. The animals were purchased from JM Instrument Supply (Selangor, Malaysia). For acclimatization, no more than three animals were housed in each Polypropylene cage, under good laboratory conditions (temperature 25 ± 3 °C; 55 ± 5% relative humidity) with 12 h dark and light cycle for minimum of 7 days. During this period they had free access to normal rat chow (Specialty Feeds, Memphis, TN, USA) and tap water *ad libitum.* At the day of dosing, all rats were weighed and examined in detail for physical abnormalities. Group assignments for those judged suitable for testing were generally based on body weight. The study includes control group administered DW and treated group administered TQRFNE. Each group consisted of 10 rats (five males and five females) and body weight ranges at the beginning of the study were 315–337 g for males and 210–243 g for females. The present study was conducted in compliance with the test Guidelines of the Organization for Economic Cooperation and Development [31], and was conducted in compliance with FDA Good laboratory practice Regulations [32]. The study protocol was approved by the Animal Care and Use Committee (Approval No. UPM/FPSK//PADS/BR-UUH/00414), Faculty of Medicine and Health Sciences, Universiti Putra Malaysia. Oral administration was selected as the route of administration using a stainless steel feeding needle, with 20 mL/kg body weight of DW or TQRFNE. Animals were fasted 12 h prior to dosing and the food was redistributed 3 h after the administration [31]. 

### 3.4. Observations

#### 3.4.1. Body Weight, and Food and Water Consumption

Body weight of each rat was taken at the initiation of treatment and once a week thereafter (days 7 and 14) using an electronic balance (Precisa Instrument, Dietikon, Switzerland). The amount of daily water and food consumption was calculated from the difference of initial amounts provided and the remnants for each group. Weekly water and food consumption/rat were also calculated based on the total consumption divided by the number of rats in each group to get the daily consumption of food (g/rat/d) and water (ml/rat/d).

#### 3.4.2. Clinical Signs and Necropsy Findings

Clinical signs and mortalities were monitored continuously for the first hour of administration periodically during the first 24 h, with special emphasis on the first 4 h, and daily thereafter, for a total of 14 days. On the necropsy day (day 15), blood samples (3 mL) were collected from overnight fasting rats into both anticoagulant-containing (EDTA) and anticoagulant-free (serum blood collection) tubes, by cardiac puncture following light anesthesia. The anti-coagulated blood samples were analyzed immediately for hematology parameters, and the anticoagulant-free blood was used for clinical biochemical study following complete clotting. The serum from the anticoagulant-free tubes was collected by centrifugation at 3500 rpm for 10 min using centrifuge machine (Hettich EBA 20, Tuttlingen, Germany), and transferred into 1.5 mL Eppendorf tubes and stored at −80 °C until analysis. Following blood collection, rats were killed immediately, and their organs (liver, kidneys, spleen, heart, lungs, brain, testes, ovaries, empty stomach and gut) were quickly removed, washed with 0.9% normal saline and weighed individually. The gross examination (macroscopic analysis) of the target organs of the controls and treated animals was conducted to monitor for any significant change in weight, texture and shape.

#### 3.4.3. ROW

Fasted body weight of each animal was measured immediately before sacrificing and the organ weights were measured within 5 min after sacrificing. The ROW (organ to body weight ratio) were calculated as (weight of organ/body weight of rat on the day of sacrifice) × 100% [33].

#### 3.4.4. Hematology

The blood was analyzed with KX-21 haematology analyzer (Kobe, Japan). WBC, RBC, HGB, HCT, MCV, MCH, MCHC, PLT, LYM, PDW, MPV and P-LCR counts were measured.

#### 3.4.5. Liver and Kidney Functions Tests

For liver and kidney function tests, serum was used to quantify the following parameters: URE, CREA, AST, ALP, and ALT using commercial assay kits (Randox analytical kits) that were based on enzymatic colorimetric methods according to the instructions of the manufacturer on an automated chemistry analyzer, Selectra XL (Vita Scientific, Dieren, The Netherlands). Results were expressed as mmol L^−1^.

#### 3.4.6. Histopathology

The liver samples collected after sacrificing the animals were washed with 0.9% normal saline and immediately placed in 10% formalin before processing. After fixation, the tissues were trimmed at 0.4–0.5 cm thickness and placed in plastic cassettes before they were processed using a tissue processor (Leica TP 1020, Nussloch, Germany). Then, the tissue samples were fixed again in 10% formalin for 1 h, dehydrated through an ethanol series (80% for 1 × 1 h, 95% for 2 × 1 h, and 100% for 3 × 1 h), washed in xylene (3 × 1 h) and paraffin embedded (2 × 2 h). Paraffin embedding was done using a Tissue Embedding Console System (Leica EG1160) via a routine method of paraffin embedding procedure. The tissue samples were sectioned at 5 μm thicknesses using a microtome (Leica RM2155) and the sections were placed on water bath (Leica H1210) at 35 °C to 37 °C. Samples were then mounted on glass slides using a hot plate (Leica HI1220) and stained with H & E stain for histological analysis.

### 3.5. Statistical Analysis

The data were recorded as mean ± standard deviation and analyzed by SPSS (version 19, SPSS Inc, Chicago, IL). Data were analyzed using one-way ANOVA, followed by Least Significant Difference (LSD). A value of *p* < 0.05 was deemed to be statistically significant.

## 4. Conclusions

In conclusion, TQRFNE was non-toxic by the oral route in male and female Sprague Dawley rats under the conditions of this study at a dose level of 20 mL/kg. The lack of toxicity of TQRFNE evidenced by the normal levels of hematological, hepatic, and renal key parameters as compared to the control groups suggests a wide margin of safety for its therapeutic doses. In addition, no hepatic toxicity was seen through the histopathology results. However, acute toxicity data sometimes is of limited clinical application since accumulative toxic effect may not be seen in short period with a single dose application. A follow-up study with long-term exposure like sub-acute and chronic toxicity studies may be needed in evaluating the safety profile of TQRFNE.
